# Genetic basis of sleep bruxism and sleep apnea—response to a medical puzzle

**DOI:** 10.1038/s41598-020-64615-y

**Published:** 2020-05-04

**Authors:** Mieszko Wieckiewicz, Katarzyna Bogunia-Kubik, Grzegorz Mazur, Dariusz Danel, Joanna Smardz, Anna Wojakowska, Rafal Poreba, Marta Dratwa, Monika Chaszczewska-Markowska, Efraim Winocur, Alona Emodi-Perlman, Helena Martynowicz

**Affiliations:** 1 Department of Experimental Dentistry, Wroclaw Medical University, 26 Krakowska St., 50–425 Wroclaw, Poland; 2 Laboratory of Clinical Immunogenetics and Pharmacogenetics, Hirszfeld Institute of Immunology and Experimental Therapy, Polish Academy of Sciences, 12 R. Weigla St., 53–114 Wroclaw, Poland; 3 Department of Internal Medicine, Occupational Diseases, Hypertension and Clinical Oncology, Wroclaw Medical University, 213 Borowska St., 50–556 Wroclaw, Poland; 4 Department of Anthropology, Hirszfeld Institute of Immunology and Experimental Therapy, Polish Academy of Sciences, 12 R. Weigla St., 53–114 Wroclaw, Poland; 5 Department of Oral Rehabilitation, The Maurice and Gabriela Goldschleger School of Dental Medicine, Tel Aviv University, 4 Klatchkin St., Tel Aviv, 69978 Israel

**Keywords:** Genetics, Neurology, Risk factors

## Abstract

Sleep bruxism (SB) and obstructive sleep apnea (OSA) are co-occurring sleep conditions. The study aimed to evaluate the association of selected single-nucleotide polymorphisms (SNPs) occurring within the genes of the serotonin and dopamine pathways in SB and OSA and investigate the relationship between them. The study group included 100 Caucasian patients. SB and OSA were diagnosed in 74 and 28 patients, respectively. In addition, 125 unrelated Caucasian healthy blood donors served as randomly selected controls to enable comparison of polymorphisms. The following SNPs were analyzed: rs2770304 and rs6313 within the serotonin receptor encoding gene *(HTR2A*), rs4680 polymorphism of the catechol-O-methyltransferase (*COMT*) gene, and rs686 within the dopamine receptor (*DRD1*) encoding gene. The prevalence of the *DRD1* rs686 *G* variant (*GG* homozygosity) was found to be high in the study group compared to the control group. Bruxism episode index (BEI) was found to be significantly increased in the *HTR2A* rs6313 *TT* homozygotes compared to the heterozygous patients. Moreover, within a group of the *HTR2A* rs2770304 *TT* homozygous cases, a statistically significant correlation was observed between BEI and apnea–hypopnea index. These results indicate that *DRD1* rs686 may potentially affect predisposition to SB, that *HTR2A* rs6313 SNP may be involved in SB pathogenesis, and that *HTR2A* rs2770304 polymorphism might contribute to the association between SB and OSA. This suggests a possible genetic contribution to the etiology of primary SB.

## Introduction

In 2013, an international group of bruxism experts issued a consensus proposal based on the concept that bruxism is “a repetitive jaw muscle activity characterized by clenching or grinding of the teeth and/or bracing or thrusting of the mandible”^1^. Bruxism can occur during sleep (sleep bruxism (SB)) or during wakefulness (awake bruxism (AB))^1^. The American Academy of Sleep Medicine initially classified SB as parasomnia overlap syndrome, but now it is considered as a movement disorder^2^. The latest international consensus on SB assessment has defined SB as” a masticatory muscle activity during sleep that is characterized as rhythmic (phasic) or nonrhythmic (tonic) and not a movement disorder or a sleep disorder in otherwise healthy individuals”^3^. The International Classification of Sleep Disorders indicates the following clinical criteria for the diagnosis of SB: “(a) the presence of regular or frequent tooth grinding sounds during sleep, and (b) the presence of one or more of the following clinical signs: (1) abnormal tooth wear consistent with the above reports of tooth grinding during sleep, and (2) transient morning jaw muscle pain or fatigue, temporal headache, and/or jaw locking upon awakening consistent with the above reports of tooth grinding during sleep”^4^. The prevalence of SB is found to be 8%–31% among the world adult population^5,6^. There is an evidence suggesting that the autonomic nervous system plays a role in the genesis of SB^7,8^. SB episodes occur in association with cortical arousal observed during electroencephalography as well as tachycardia, which is associated with transient elevations of sympathetic tone^9,10^, suggesting involvement of neurotransmitters in the pathogenesis of SB. Some studies have reported correlation between central dopaminergic^11,12^ or serotoninergic mechanisms^13^ and bruxism.

Obstructive sleep apnea (OSA) is a common sleep disorder characterized by “repetitive collapse of the upper airway leading to partial or complete cessation of airflow, intrathoracic pressure changes, and arterial oxygen desaturation, often terminated by an arousal from sleep resulting in muscle activation and recovery of airway patency”^14^. The prevalence of OSA is found to be approximately 22% in men and 17% in women^15^. This disorder is independently associated with hypertension^16^, stroke^17^, myocardial ischemia^18^, and arrhythmias with an increased risk for sudden cardiac death^19^. Excessive daytime sleepiness, chronic fatigue, and decreased quality of life are also consequences of OSA^20^. Recently, OSA has been considered as a new risk factor for SB^21^. An association between SB and OSA has been reported in earlier works^22,23^; however, data concerning this relationship are inconsistent^24^.

Both SB and OSA are probably genetically complex conditions that are likely to result from multiple interactions between genetic and environmental factors^25^. OSA is a heritable and there is evidence indicating direct contributions of genetic factors to OSA susceptibility and also indirect contributions via “intermediate” phenotypes such as obesity, craniofacial structure, neurological control of upper airway muscles and sleep, and circadian rhythm^26^. Polysomnographic data show that 37% of the SB subjects had at least one first-degree relative with reported SB^27^. Smoking, use of certain medications, and breathing problems can be considered as risk factors for SB^28^; however, there is controversy regarding the influence of hereditary factors, and data are inconclusive.

The aim of the study was (a) to evaluate four single-nucleotide polymorphisms (SNPs) within the genes coding for catechol-O-methyltransferase (COMT), and serotonin (HTRA2) and dopamine (DRD1) receptors in a group of patients with SB or OSA and controls; (b) to evaluate their effect on SB; and (c) to evaluate their effect on the relationship between OSA and SB. The selected SNPs may have some functional implications and may play a regulatory role, as they were reported to be associated with differential expression of an encoded protein (as shown for the *HTR2A* rs6313 SNP)^29–31^, cause structural differences (the *COMT* rs4680 SNP), or result in various mRNA interactions with microRNA molecules (the *DRD1* rs686 SNP)^32^. Both the selected *HTR2A* SNPs (rs6313 and rs2770304) have been previously studied in SB and were found to be associated with the entity in patients from Japan^13,33^ and Chile^34^ (rs6313 and rs2770304, respectively). To the best of our knowledge, only these two SNPs were reported to be associated with SB, however, not in European populations. Thus, our present study can also potentially verify these previous observations in Europeans, specifically in our Polish population.

## Results

The sample size of the study is large compared to other studies of this type^13,33,34^ considering the need for a polysomnographic study and performance of genetic tests. Consequently, the significance of results is exploratory and not confirmative.

### Distribution of SNP alleles and genotypes in patients and controls

Minor allele frequencies (MAFs) of all the studied SNPs in Polish patients and controls are presented in Table 1. MAF values in the healthy control group of our current study did not differ from those reported previously for Europeans, taken from the NCBI website (https://www.ncbi.nlm.nih.gov/snp) (C = 0.33 vs 0.34, G = 0.48 vs 0.50, T = 0.39 vs 0.44, G = 0.35 vs 0.40, for minor allele counts of rs2770304, rs4860, rs63131, and rs686 polymorphisms, respectively).

**Table 1:** Minor allele frequencies (MAFs) of the polymorphisms studied in Polish patients with sleep bruxism (SB) and healthy individuals. Comparison of the *HTR2A* rs6313 and rs2770304 MAFs in Japanese^13^, Chilean^34^, and Polish SB patients and controls.

Polymorphism/population	Minor allele	MAF in SB patients	MAF in Controls	References
***Japanese***				
*HTR2A* rs6313	*T*	0.462	0.615^c,d^	Abe *et al*.^13^
*HTR2A* rs2770304	*C*	0.462^a^	0.313	Abe *et al*.^13^
***Chilean***				
*HTR2A* rs6313	*T*	0.46	0.41^d^	Oporto *et al*.^34^
*HTR2A* rs2770304	*C*	0.44^b^	0.27	Oporto *et al*.^34^
***Polish***				
*HTR2A* rs6313	*T*	0.465	0.388^c^	Present study
*HTR2A* rs2770304	*C*	0.235^a,b^	0.332	Present study
*COMT* rs4680	*G*	0.480	0.484	Present study
*DRD1* rs686^^^	*G*	0.460^^^	0.352^^^	Present study

Notes: ^a^0.462 vs 0.235, p < 0.0001; ^b^0.44 vs 0.235, p < 0.004.

^c^0.615 vs 0.388, p < 0.0002; ^d^0.615 vs 0.41, p < 0.004.

^p = 0.0258.

All the SNPs did not show deviation from the Hardy–Weinberg equilibrium, either in patients or in controls (all p-values >0.05).

Comparison of the SNP genotypes and allele frequencies (rs2770304, rs4860, and rs63131) between our study group and healthy subjects did not show significant differences. Interestingly, a higher representation of the *DRD1* rs686 *GG* homozygosity was seen among patients as compared to controls (18% vs 8%, OR = 2.524, p = 0.0267, Table 2), suggesting a potential association of this SNP with phenomenon predisposition. Also, comparing the allelic frequencies of this SNP, a prevalence of the *G* allele was observed (p = 0.0258, Table 1).

**Table 2:** Distribution of the *HTR2A* (rs6313 and rs2770304), *COMT* (rs4680), and *DRD1* (rs686) genotypes in study group and healthy controls.

Polymorphism	Study group, n (%)	Controls, n (%)	OR, p-value
***HTR2A*** **rs6313**			
*CC*	31 (31.3%)	47 (37.6%)	ns
*CT*	44 (44.4%)	59 (47.2%)	ns
*TT*	24 (24.3%)	19 (15.2%)	ns
***HTR2A*** **rs2770304**			
*TT*	59 (59.0%)	59 (47.2%)	ns
*TC*	35 (35.0%)	49 (39.2%)	ns
*CC*	6 (6.0%)	17 (13.6%)	ns
***COMT*** **rs4680**			
*GG*	23 (23.0%)	26 (20.8%)	ns
*GA*	50 (50.0%)	69 (55.2%)	ns
*AA*	27 (27.0%)	30 (24.0%)	ns
***DRD1*** **rs686**			
*AA*	26 (26.0%)	47 (37.6%)	OR = 0.599, p = 0.0852
*AG*	56 (56.0%)	68 (54.4%)	ns
*GG*	18 (18.0%)	10 (8.0%)	OR = 2.524, p = 0.0267

Notes: n—number of patients with a given genotype, ns—nonsignificant.

These relationships of the *DRD1* rs686 SNP with SB risk have not been previously described. However, two other SNPs, the *HTR2A* rs6313 and rs2770304 polymorphisms, have been reported to be associated with SB risk in patients from Japan^13,33^ and Chile^34^, respectively. The observed differences in MAF values between the Polish, Japanese, and Chileans (Table 1) may, at least in part, explain the various associations with predisposition to the disease found in these three populations.

### Associations between SNPs and clinical parameters—bruxism episode index (BEI) and apnea–hypopnea index (AHI)

Descriptive statistics for clinical parameters are presented in Table 3.

**Table 3:** Descriptive statistics of the SB patients’ data (M: arithmetic mean, SD: standard deviation, Me: median).

HTR2A rs6313		N	BEI			logBEI			AHI			Phasic			Tonic			Mixed		
			M	SD	Me	M	SD	Me	M	SD	Me	M	SD	Me	M	SD	Me	M	SD	Me
CC	Women	21	3.82	2.14	3.70	1.14	0.73	1.31	2.00	1.88	1.20	1.78	1.51	1.10	1.21	0.88	1.10	0.90	0.60	0.70
	Men	10	8.48	7.20	7.85	1.71	1.10	2.06	11.01	16.91	3.90	5.44	5.94	4.00	1.76	1.36	1.40	1.16	0.86	1.20
	All	31	5.32	4.85	4.30	1.33	0.89	1.46	4.90	10.3	1.90	2.96	3.89	1.70	1.39	1.07	1.30	0.98	0.69	0.90
CT	Women	32	3.72	2.96	2.95	0.94	0.98	1.08	3.99	7.08	1.60	1.23	1.47	0.80	1.59	1.11	1.35	0.97	0.99	0.75
	Men	12	3.13	2.41	2.25	0.85	0.80	0.78	9.94	13.98	3.65	0.61	0.75	0.30	1.83	1.31	1.30	0.70	0.79	0.35
	All	44	3.56	2.81	2.80	0.92	0.92	1.03	5.62	9.7	1.95	1.06	1.34	0.60	1.65	1.16	1.35	0.90	0.94	0.65
TT	Women	15	5.30	4.43	3.90	1.33	0.95	1.36	5.48	8.62	2.40	2.05	2.70	0.60	2.10	1.78	1.80	1.21	0.98	1.10
	Men	9	6.69	3.35	7.00	1.70	0.78	1.95	13.30	10.20	9.40	1.77	1.49	2.20	3.48	2.14	3.00	1.77	1.44	1.30
	All	24	5.82	4.04	5.50	1.47	0.89	1.70	8.41	9.8	4.90	1.95	2.29	0.75	2.62	1.99	2.15	1.42	1.17	1.10
All groups		99	4.66	3.94	3.50	1.18	0.93	1.25	6.07	9.9	2.10	1.87	2.70	0.90	1.81	1.45	1.40	1.05	0.95	0.80
**HTR2A rs2770304**		**N**	**BEI**	**logBEI**	**AHI**	**Phasic**	**Tonic**	**Mixed**												
			**M**	**SD**	**Me**	**M**	**SD**	**Me**	**M**	**SD**	**Me**	**M**	**SD**	**Me**	**M**	**SD**	**Me**	**M**	**SD**	**Me**
CC	Women	4	3.88	3.03	3.50	1.06	0.93	1.12	2.25	2.16	2.40	2.20	2.00	1.90	1.13	0.70	1.35	0.60	0.71	0.35
	Men	2	5.15	4.45	5.15	1.40	1.01	1.40	2.40	0.71	2.40	3.70	4.53	3.70	1.05	0.35	1.05	0.60	0.57	0.60
	All	6	4.30	3.15	3.60	1.18	0.87	1.17	2.30	1.71	2.40	2.70	2.67	1.90	1.10	0.57	1.30	0.60	0.60	0.40
TT	Women	34	4.80	3.52	3.95	1.27	0.90	1.37	3.35	5.99	1.55	1.84	2.13	0.85	1.91	1.52	1.55	1.14	0.79	1.05
	Men	25	5.87	5.05	5.40	1.42	0.90	1.69	13.35	15.43	6.60	2.15	3.84	1.20	2.52	1.77	2.00	1.23	1.09	0.90
	All	59	5.26	4.23	4.20	1.34	0.90	1.44	5.42	9.32	1.60	1.97	2.95	0.90	2.17	1.64	1.70	1.18	0.92	1.00
TC	Women	30	3.33	2.61	2.75	0.88	0.88	1.01	4.30	7.33	1.30	1.21	1.35	0.80	1.28	0.83	1.10	0.89	0.98	0.60
	Men	5	6.00	6.18	2.80	1.16	1.38	1.03	12.10	16.83	3.60	3.44	4.86	0.40	1.76	1.57	1.00	0.90	1.26	0.20
	All	35	3.71	3.34	2.80	0.92	0.94	1.03	7.59	11.99	2.50	1.53	2.23	0.80	1.35	0.95	1.10	0.89	1.00	0.60
All groups		100	4.66	3.92	3.60	1.18	0.92	1.28	6.51	10.79	2.20	1.86	2.69	0.85	1.82	1.45	1.40	1.05	0.95	0.80
**COMT rs4680**		**N**	**BEI**	**logBEI**	**AHI**	**Phasic**	**Tonic**	**Mixed**												
			**M**	**SD**	**Me**	**M**	**SD**	**Me**	**M**	**SD**	**Me**	**M**	**SD**	**Me**	**M**	**SD**	**Me**	**M**	**SD**	**Me**
GA	Women	36	4.37	3.16	3.60	1.17	0.91	1.28	3.26	5.84	1.60	1.68	2.00	0.80	1.67	1.01	1.45	1.13	1.02	0.85
	Men	14	5.83	4.15	6.95	1.42	0.96	1.94	9.89	11.45	5.45	2.61	3.23	1.90	2.29	1.53	1.85	1.21	1.07	1.05
	All	50	4.78	3.48	3.80	1.24	0.92	1.34	5.11	8.26	2.20	1.94	2.41	0.85	1.84	1.19	1.45	1.15	1.03	0.95
GG	Women	11	4.12	1.94	4.10	1.29	0.54	1.41	4.02	8.73	1.20	1.76	1.48	1.30	1.40	1.14	1.00	0.99	0.63	1.10
	Men	12	6.09	6.95	4.05	1.23	1.16	1.34	8.63	10.21	4.25	2.53	5.49	0.35	2.44	2.28	1.25	0.93	1.20	0.40
	All	23	5.15	5.18	4.10	1.26	0.90	1.41	6.43	9.61	2.40	2.16	4.02	0.80	1.94	1.86	1.00	0.96	0.95	0.70
AA	Women	21	3.62	3.70	2.00	0.85	1.01	0.69	4.31	6.34	1.60	1.32	1.72	0.60	1.55	1.67	1.10	0.78	0.70	0.50
	Men	6	5.40	2.47	4.50	1.61	0.43	1.50	26.17	23.77	21.60	1.90	1.41	1.30	2.12	0.74	1.90	1.40	0.92	1.10
	All	27	4.02	3.50	3.40	1.02	0.96	1.22	9.17	15.01	2.10	1.45	1.65	0.90	1.67	1.52	1.30	0.92	0.78	0.70
All groups		100	4.66	3.92	3.60	1.18	0.92	1.28	6.51	10.79	2.20	1.86	2.69	0.85	1.82	1.45	1.40	1.05	0.95	0.80
**DRD1 rs686**		**N**	**BEI**	**logBEI**	**AHI**	**Phasic**	**Tonic**	**Mixed**												
			**M**	**SD**	**Me**	**M**	**SD**	**Me**	**M**	**SD**	**Me**	**M**	**SD**	**Me**	**M**	**SD**	**Me**	**M**	**SD**	**Me**
AA	Women	17	4.45	2.48	3.90	1.28	0.81	1.36	3.47	6.03	2.00	1.64	1.75	1.10	1.86	1.18	1.50	0.96	0.54	0.80
	Men	9	7.32	7.44	5.40	1.51	1.12	1.69	18.33	13.45	17.20	2.98	6.22	0.50	2.63	2.02	2.30	1.43	1.45	0.90
	All	26	5.44	4.86	4.05	1.36	0.92	1.40	8.62	11.5	2.65	2.10	3.84	0.70	2.13	1.53	1.70	1.12	0.95	0.85
AG	Women	37	3.70	2.72	3.10	1.00	0.88	1.13	3.92	6.62	1.50	1.46	1.44	0.80	1.44	1.01	1.30	0.89	0.94	0.60
	Men	19	5.84	3.90	6.90	1.45	0.92	1.93	9.83	15.74	3.70	2.46	2.96	1.60	2.39	1.70	2.00	1.05	0.98	0.60
	All	56	4.43	3.30	3.40	1.15	0.91	1.22	5.93	10.9	2.05	1.80	2.11	0.80	1.76	1.35	1.30	0.94	0.95	0.60
GG	Women	14	4.74	4.71	3.20	1.11	1.07	1.13	3.42	6.88	1.15	1.84	2.74	0.75	1.66	1.83	1.15	1.34	1.01	1.20
	Men	4	2.55	1.61	2.35	0.77	0.69	0.78	11.83	15.48	5.75	1.20	0.91	1.30	1.20	0.43	1.10	0.90	0.52	1.05
	All	18	4.25	4.27	2.80	1.04	0.99	1.02	5.29	9.6	1.30	1.69	2.44	0.90	1.56	1.62	1.10	1.24	0.93	1.20
All groups		100	4.66	3.92	3.60	1.18	0.92	1.28	6.51	10.8	2.20	1.86	2.69	0.85	1.82	1.45	1.40	1.05	0.95	0.80

**Figure 1:**
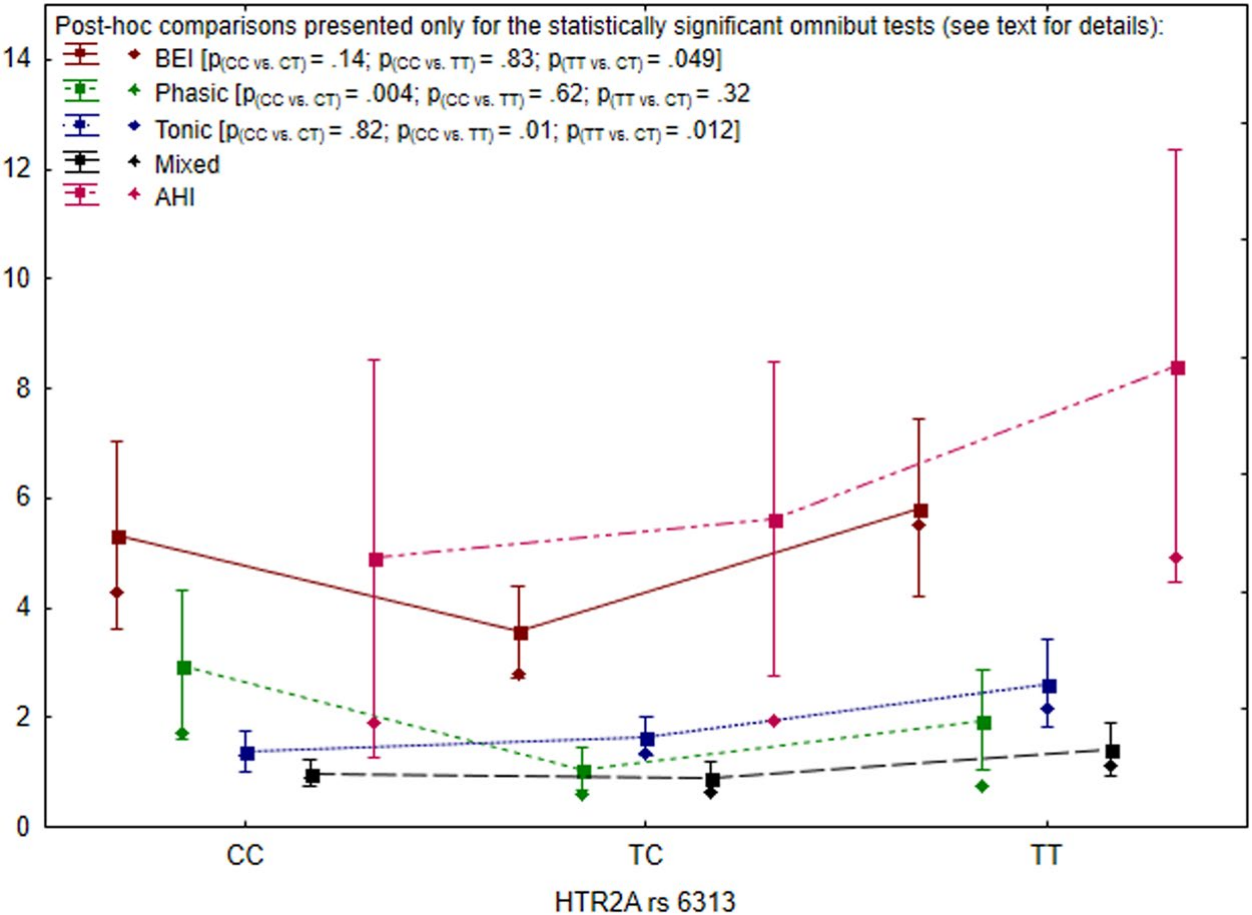
Clinical parameters and SNPs for *HTR2A* rs6313 (rectangles = arithmetic means, rhombuses = medians, whiskers = 1.96 × standard error).

The effect of *HTR2A* rs6313 polymorphism on logBEI values was statistically significant [F (2,96) =3.43, p = 0.036, η^2^ = 0.07]. Post-hoc pairwise comparisons showed that the mean logBEI values were significantly higher in *TT* homozygous patients when compared to the *TC* carriers (p = 0.049). The mean logBEI values were not found to be statistically different in *CC* and *TT* homozygotes, and between *CC* and *TC* patients (Table 4, Fig. 1).

**Table 4:** Spearman’s rank correlations between BEI and AHI values in SB patients in relation with the gene polymorphisms.

	n	r_s_	p
***HTR2A*** **rs6313**			
*CC*	31	0.20	0.29
*CT*	44	0.13	0.39
*TT*	24	0.37	0.07
***HTR2A*** **rs2770304**			
*CC*	6	0.14	0.79
*TT*	59	0.32	0.01
*TC*	35	–0.08	0.65
***COMT*** **rs4680**			
*GA*	50	0.25	0.08
*GG*	23	0.07	0.76
*AA*	27	0.27	0.17
***DRD1*** **rs686**			
*AA*	26	0.26	0.19
*AG*	56	0.11	0.40
*GG*	18	0.11	0.66

The values of phasic SB episodes were statistically significantly different among the three groups of patients [H (2, N = 99)=10.60, p = 0.005, η^2^ = 0.09]. Post-hoc analysis showed one statistically significant difference—when compared to the *TC* patients, *CC* individuals had higher intensity of phasic episodes (p = 0.004). Similarly, gene polymorphism significantly differentiated the occurrence of tonic episodes in the three analyzed genotypes [H (2, N = 99) = 8.27, p = 0.016, η^2^ = 0.07]. Post-hoc analysis showed that the differences were statistically significant only between *CC* and *TT* carriers (p = 0.013). The group differences for mixed episodes were found to be statistically nonsignificant [H (2, N = 99) = 4.21, p = 0.12] (Fig. 1).

The differences in the logBEI values were statistically nonsignificant for the *HTR2A* rs2770304 genotypes [F (2,97) = 2.26, p = 0.11]. Similarly, for the same SNP, there were no statistically significant group differences in the phasic episodes [H (2, N = 100)=2.14, p = 0.34].

**Figure 2:**
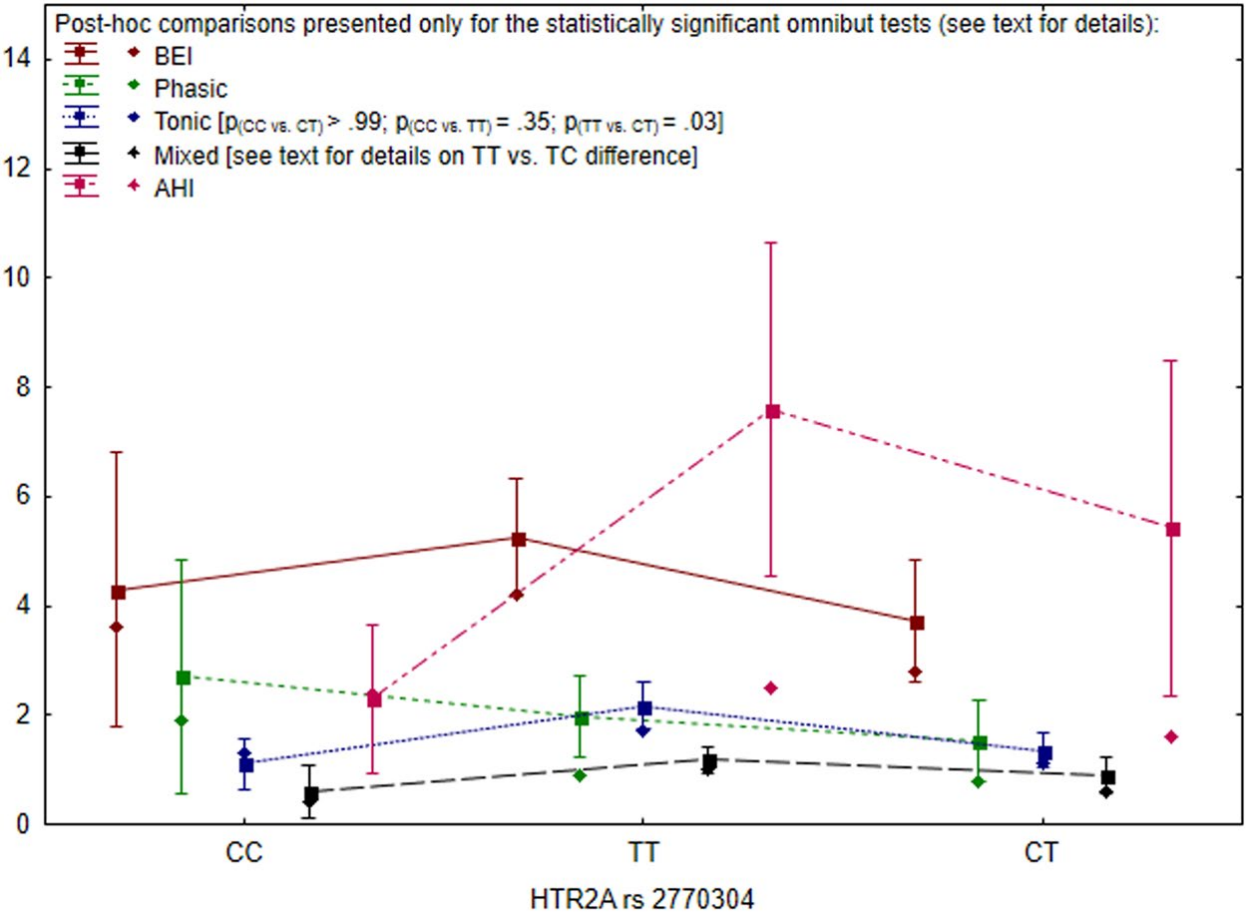
Clinical parameters and SNPs for *HTR2A* rs2770304 (rectangles = arithmetic means, rhombuses = medians, whiskers = 1.96 × standard error).

However, the *HTR2A* rs2770304 polymorphism significantly differentiated the tonic values [H (2, N = 100)=7.98, p = 0.02, η^2^ = 0.06]. Detailed post-hoc pairwise comparisons showed that tonic values were significantly higher in the *TT* patients when compared to the *TC* individuals (p = 0.03). Other pairwise comparisons (*CC* vs *TC* and *CC* vs *TT*) were statistically nonsignificant. The differences between the intensity of mixed episodes shown by different genotypes of *HTR2A* rs2770304 were marginally significant [H (2, N = 100) = 5.65, p = 0.06, η^2^ = 0.04]. The post-hoc pairwise comparisons did not reveal any statistically significant group differences. However, the difference between mixed values, highest in the *TT* individuals and lowest in the *TC* patients, reached the statistical significance level after excluding the smallest group (n = 6) of *CC* patients from the analysis [H(1, N = 94) = 3.88, p = 0.05, η^2^ = 0.03]. Detailed results for *HTR2A* rs2770304 polymorphism are presented Fig. 2.

**Figure 3:**
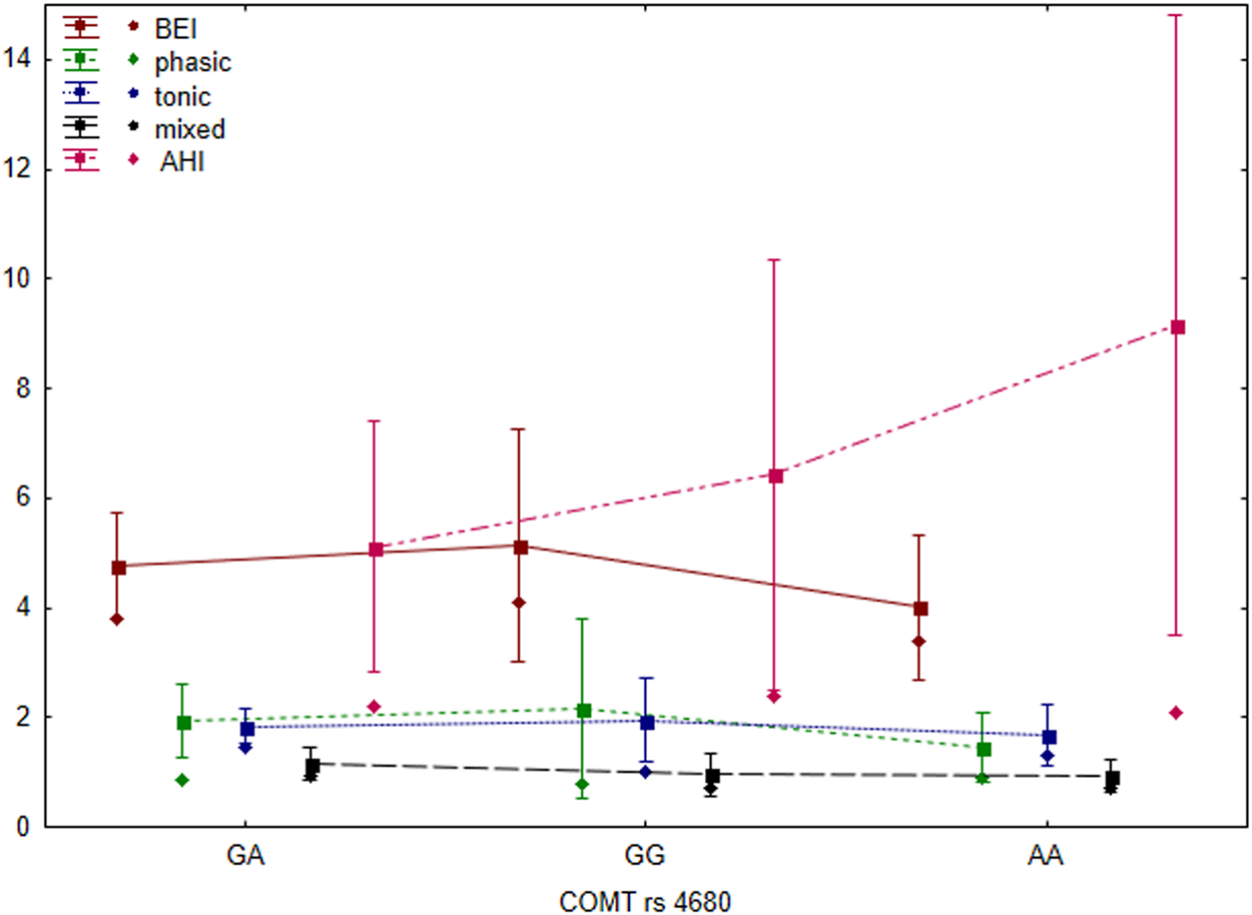
Clinical parameters and SNPs for *COMT* rs4680 (rectangles = arithmetic means, rhombuses = medians, whiskers = 1.96 × standard error).

**Figure 4:**
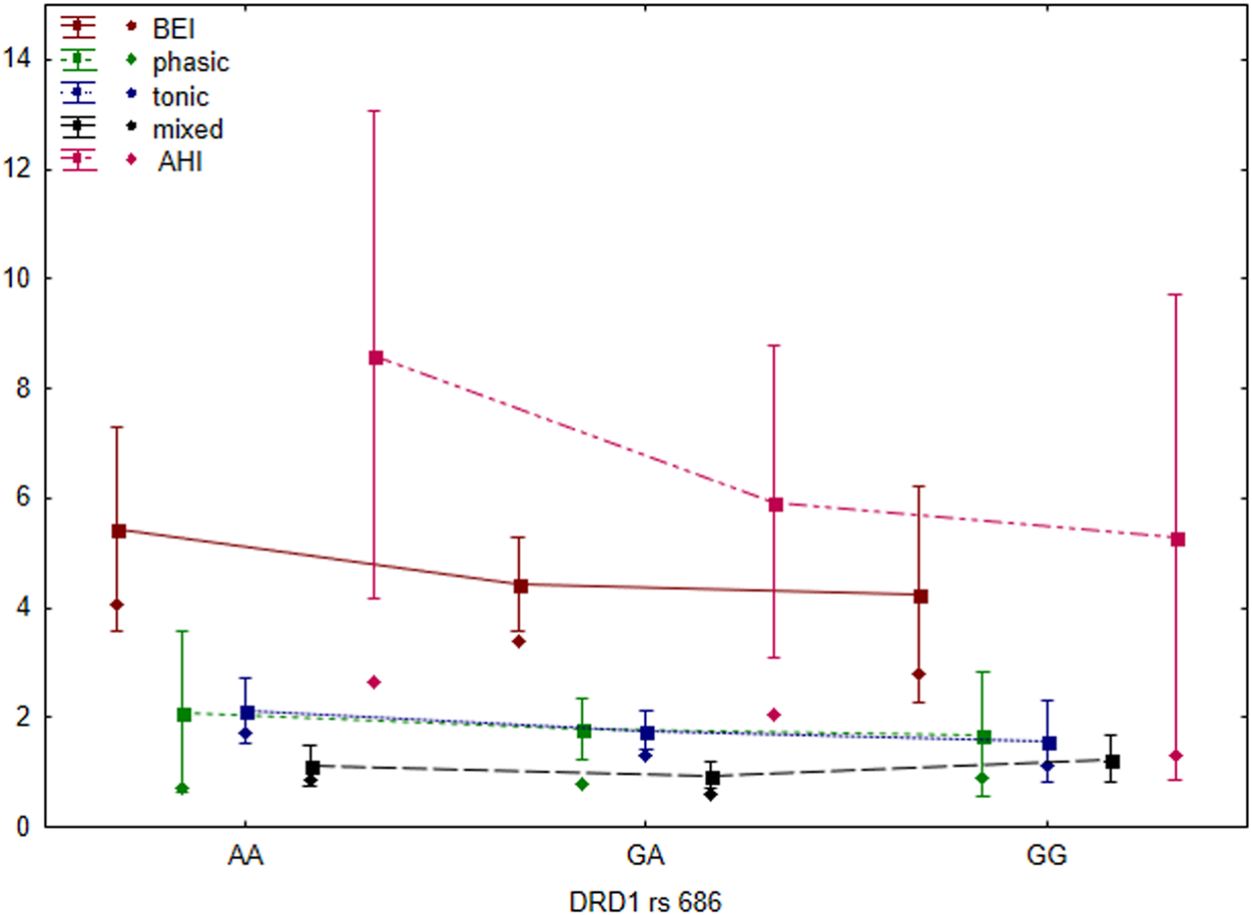
Clinical parameters and SNPs for *DRD1* rs686 (rectangles = arithmetic means, rhombuses = medians, whiskers = 1.96 × standard error).

Neither *COMT* rs4680 nor *DRD1* rs686 polymorphism showed significant difference in the logBEI values. Similarly, for both polymorphisms, statistically nonsignificant differences were observed in the measures of tonic, phasic, and mixed bruxism episodes between *GA*, *AA*, and *GG* patients (results are presented in Table 4). Moreover, the differences in the AHI values were statistically nonsignificant for all the analyzed SNPs (results are presented in Figs. 3 and 4).

Finally, we analyzed the relationship between BEI and AHI scores. The correlation between BEI and AHI values were statistically significant only in patients homozygous for the *HTR2A* rs2770304 T allele. In this group of patients, increase in the BEI values was related to higher AHI values (rs(59)=0.32, p = 0.01). The correlations in *HTR2A* rs6313 *TT* and *COMT* rs4680 *GA* patients were found to be marginally significant.

### Clinical relevance of the polymorphism for OSA, SB and co-occurring OSA and SB

In the last stage of the statistical analysis, we assessed clinical relevance of the observed polymorphism for OSA only, SB only and co-occurring OSA and SB patients diagnosed in the study group. Detailed results are summarized in Table 5.

**Table 5:** Clinical relevance of detected polymorphisms assessed by Sensitivity, Specificity, Negative Predictive Value (NPV) and Positive Predicted Value (PPV).

	Sensitivity	Specificity	PPV	NPV
**SB only**				
HTR2A rs6313				
CC	42.31	77.78	78.57	41.18
CT	40.38	66.67	70.00	36.73
TT	17.31	55.56	42.86	25.86
HTR2A rs2770304				
CC	7.69	100.00	100.00	36.84
CT	32.69	67.86	65.38	35.19
TT	59.62	32.14	62.00	30.00
COMT rs4680				
GA	57.69	60.71	73.17	43.59
GG	25.00	78.57	68.42	36.07
AA	17.31	60.71	45.00	28.33
DRD1 rs686				
GA	55.77	50.00	67.44	37.84
GG	19.23	85.71	71.43	36.36
AA	25.00	64.29	56.52	31.58
**OSA only**				
HTR2A rs6313				
CC	33.33	64.38	7.14	92.16
CT	50.00	63.01	10.00	93.88
TT	16.67	72.60	4.76	91.38
HTR2A rs2770304				
CC	0.00	94.59	0.00	92.11
CT	66.67	70.27	15.38	96.30
TT	33.33	35.14	4.00	86.67
COMT rs4680				
GA	33.33	47.30	4.88	89.74
GG	33.33	77.03	10.53	93.44
AA	33.33	75.68	10.00	93.33
DRD1 rs686				
GA	66.67	47.30	9.30	94.59
GG	16.67	82.43	7.14	92.42
AA	16.67	70.27	4.35	91.23
**Co-occurring SB and OSA**				
HTR2A rs6313				
CC	19.05	58.62	14.29	66.67
CT	28.57	58.62	20.00	69.39
TT	52.38	82.76	52.38	82.76
HTR2A rs2770304				
CC	0.00	93.10	0.00	71.05
CT	22.73	63.79	19.23	68.52
TT	77.27	43.10	34.00	83.33
COMT rs4680				
GA	40.91	44.83	21.95	66.67
GG	18.18	74.14	21.05	70.49
AA	40.91	81.03	45.00	78.33
DRD1 rs686				
GA	45.45	43.10	23.26	67.57
GG	13.64	81.03	21.43	71.21
AA	40.91	75.86	39.13	77.19

## Discussion

In the presented study the following SNPs were analyzed: rs2770304 and rs6313 within the serotonin receptor encoding gene (HTR2A), rs4680 polymorphism of the catechol-O-methyltransferase (COMT) gene, and rs686 within the dopamine receptor (DRD1) encoding gene. The most important results obtained in this study showed that the prevalence of the DRD1 rs686 G variant (GG homozygosity) was found to be high in the study group compared to the control group, suggesting a potential association of this SNP with studied condition predisposition. BEI was found to be significantly increased in the HTR2A rs6313 TT homozygotes compared to the heterozygous patients. Moreover, within a group of the HTR2A rs2770304 TT homozygous cases, a statistically significant correlation was observed between BEI and AHI.

Since it is known that central nervous system plays a role in the pathogenesis of SB, the role of brain neurotransmitters, including dopamine and serotonin, was investigated in a few studies^8,9,13^. The serotonin receptor HTRA2 plays an important role in mood regulation in adults, and the *HTR2A* gene polymorphisms are found to be associated with a number of psychiatric disorders^35^. Serotonin 2A receptors are concentrated in the limbic system, and hence they are of importance for mediating the emotion of fear. Moreover, expression of these receptors constitutes a trait related to anxiety^36^. It was shown that bruxers showed significant differences in anxiety, hostility, and phobic anxiety compared to non-bruxers^37^. A controlled laboratory study reported that SB patients were more competitive and felt more anxious than normal subjects^38^, thus serotonin pathway may be considered in SB pathogenesis. It is a well-known fact that long-term usage of selective serotonin reuptake inhibitors may cause bruxism^39^. Abe *et al*. showed that the *C* allele of the *HTR2A* rs6313 SNP was significantly associated with an increased risk of SB, and thus authors of this study concluded that it may contribute to the etiology of the condition^13^. However, polysomnography was not used in this study and diagnosis was based on masseter electromyographic recordings, performed by using a portable miniature device, and clinical symptoms^13^. Hoashi *et al*. continued this research work by using polysomnography to diagnose SB; however, only two bruxers and two non-bruxers were investigated in this study^33^. Also, Oporto *et al*. showed that the *HTR2A* rs2770304 SNP is involved in the occurrence of SB^34^. Therefore, we decided to examine a group of patients (n = 100) using video polysomnography, a gold standard for SB assessment, to evaluate the effect of selected polymorphisms of genes involved in serotonin and dopamine pathways on SB. The *HTR2A* gene is located on chromosome 13q14-q21. Both polymorphisms within the *HTR2A* gene (rs6313 and rs2770304 within exon 1 and intron 2, respectively), previously found to affect the development of SB, were investigated.

No significant difference was detected between the studied patients and controls with respect to the distribution of the *HTRA2* alleles and genotypes. Lack of association with the *HTRA2* SNPs might be a result of inter-population differences, and indeed (as shown in Table 1), patients and controls of the present study did show significantly different MAF values than the Japanese and Chileans.

However, some significant differences were noticed when patients were stratified with respect to the rs6313 SNP of the *HTR2A* gene. The comparative analysis showed that homozygotes for allele *T* (*TT*) had statistically significantly increased BEI score compared to heterozygotes (*CT*). Interestingly, when patients with various types of bruxism (tonic, phasic, and mixed) were considered, the analysis showed that the incidence of tonic BEI was increased in the group comprising *HTR2A* rs6313 *CT* heterozygotes compared to homozygotes for allele *C* (*CC*). On the contrary, in the group including patients with phasic bruxism, the BEI was increased in the *HTR2A* rs6313 *CC* homozygous patients compared to the *CT* heterozygotes. We have also observed increased tonic BEI in the group of heterozygotes (*TC*) compared to homozygotes for allele *C* of the *HTR2A* rs2770304 SNP. Thus, these results underline and confirm the significance of the serotonin pathway in the pathogenesis of SB and show the associations between SNPs within the *HTRA2* gene and BEI values.

Serotonin is a neurotransmitter responsible for maintaining the circadian rhythm, controlling arousal level, and regulating stress response, food intake, sleep, anxiety, sexual behavior, mood, muscle tone, and also breathing^40^. Four serotonin receptors, HTR1B, SLC6A4, HTR2A, and HTR2C, were found to be the potential candidates for OSA regulation^41^; however, the results of previous studies have been inconsistent. We also did not find any significant association of the AHI values with any allele or genotype of the studied polymorphisms. However, it has to be emphasized that we did observe a correlation between BEI and AHI in the group of patients homozygous for allele *T* of the rs2770304 SNP in the *HTR2A* gene. The results of our study may explain the contradicting data of the previous studies investigating the correlation between OSA and SB. The occurrence of this correlation may depend on a studied population. The correlation may be observed if allele *T* of the SNP rs2770304 of the *HTR2A* gene is predominant. If allele *C* prevails in a studied population, the attempt to find a correlation will fail.

We also investigated the importance of dopamine pathway genes in the pathogenesis of SB. Dopamine is involved in motor control, cognition, pain perception^42^, and the reward system^43^ of central nervous system^44^. Because of similarities to the restless legs syndrome, a link to changes in central dopamine activity has been considered in both conditions. The *COMT* gene, located on chromosome 22 (22q11), is involved in the extracellular degradation of catecholamines (dopamine, norepinephrine, and epinephrine). The functional *COMT* Val158Met polymorphism has been related to different levels of enzyme activity. This SNP is associated with risk of depression^45^, schizophrenia^46^, pain sensitivity^47^, and complex emotion recognition^48^, and it may also affect the other conditions. The dopamine receptor D1 (DRD1), encoded by the *DRD1* gene located on chromosome 5q35.1, is a member of the D1 subfamily of dopaminergic receptors^49^. This receptor is involved in social cognition, executive functioning, working memory, and neuropsychiatric disorders, such as alcohol dependence and pathological gambling^50,51^ or depressive symptoms^32^. The use of L-dopa, a dopamine precursor^52^, and bromocriptine, a D2 receptor agonist^53^, inhibits bruxism activity in polysomnographic studies. Recently, Cahlin *et al*. reported that the dopaminergic agent pramipexole did not affect SB^53^. It is important to note that polysomnography was used in the described study, which is the only method to unequivocally detect SB^54,55^.

The functional *COMT* SNP was not found to be associated with SB risk and/or BEI or AHI values in the present study. However, our results might suggest the association of the *DRD1* rs686 polymorphism with SB risk. We observed that the presence of the *DRD1* rs686 GG homozygosity resulted in a twofold increase in the risk for SB. The rs686 SNP is located within the 3′-untranslated region (3′UTR) of the *DRD1* gene and thus can be affected by microRNA (miR) molecules that can regulate its expression, and indeed, it has been documented that it is situated within the miR-504 binding site^56^. In a luciferase assay, it was found that this polymorphism leads to allele-specific differential expression of the *DRD1* gene, with the *G* allele showing a lower luciferase activity in comparison to the *A* allele^57,58^.

The effect of this observed relationship obviously warrants further studies, including confirmatory genotyping studies involving a higher number of cases from various populations as well as some functional analysis with the miRNA-504 molecule.

## Conclusions

Our findings suggest a possible genetic contribution of the variability within the serotonin receptor encoding gene (*HTR2A*) and possibly also within the dopamine (*DRD1*) receptor gene to the etiology of SB. The *DRD1* rs686 polymorphism seems to potentially affect the risk for SB development, the rs6313 *HTR2A* SNP is involved in the pathogenesis of SB, while the *HTR2A* rs2770304 polymorphism may affect the relationship of SB with OSA. Although, the obtained results are probably not sufficient to certainly link prevalence of SB with entire serotonin and dopamine pathways, they seem to be very promising and are an excellent foundation for further research.

## Materials and methods

The study group included 100 Caucasian patients (69 females and 31 males, mean age 35.2 ± 11.41 years, range 18–70 years) visiting the Department of Internal Medicine, Occupational Diseases, Hypertension and Clinical Oncology at the Wroclaw Medical University, and hospitalized for probable SB. In addition, 125 unrelated, matched for age Caucasian healthy blood donors served as randomly selected controls to enable comparison of polymorphisms (62 females and 63 males, mean age 29.98 ± 9.23 years, range 19–64 years).

Patients were enrolled between March 2017 and April 2018 by qualified dentists in the Clinic of Prosthetic Dentistry operating at the Department of Prosthetic Dentistry, Wroclaw Medical University, Poland. Sleep bruxism was diagnosed based on polysomnographic studies, and 74% (n = 74) of the patients were found to suffer from this entity, which included mild bruxism (n = 28) and severe bruxism (n = 46). OSA was diagnosed in 28% (n = 28) of the cases, and was classified as mild (n = 14), moderate (n = 8), and severe (n = 6) OSA. 22% (n = 22) had co-occurring SB and OSA. Detailed data on genotypes and clinical characteristic of the study group are presented in Table 6.

**Table 6:** Genotypes and clinical characteristic of the study group.

		SB only	OSA only	SB and OSA	Non-clinical
**HTR2A rs 6313**					
CC	Number	22	2	4	3
	% column	42,31%	33,33%	19,05%	15,00%
	% row	70,97%	6,45%	12,90%	9,68%
	% whole sample	22,22%	2,02%	4,04%	3,03%
TC	Number	21	3	6	14
	% column	40,38%	50,00%	28,57%	70,00%
	% row	47,73%	6,82%	13,64%	31,82%
	% whole sample	21,21%	3,03%	6,06%	14,14%
TT	Number	9	1	11	3
	% column	17,31%	16,67%	52,38%	15,00%
	% row	37,50%	4,17%	45,83%	12,50%
	% whole sample	9,09%	1,01%	11,11%	3,03%
Total N		52	6	21	20
Total %		52,53%	6,06%	21,21%	20,20%
**HTR2A rs2770304**					
CC	Number	4	0	0	2
	% column	7,69%	0,00%	0,00%	10,00%
	% row	66,67%	0,00%	0,00%	33,33%
	% whole sample	4,00%	0,00%	0,00%	2,00%
TT	Number	31	2	17	9
	% column	59,62%	33,33%	77,27%	45,00%
	% row	52,54%	3,39%	28,81%	15,25%
	% whole sample	31,00%	2,00%	17,00%	9,00%
CT	Number	17	4	5	9
	% column	32,69%	66,67%	22,73%	45,00%
	% row	48,57%	11,43%	14,29%	25,71%
	% whole sample	17,00%	4,00%	5,00%	9,00%
Total N		52	6	22	20
% whole		52,00%	6,00%	22,00%	20,00%
COMT rs4680					
GA	Number	30	2	9	9
	% column	57,69%	33,33%	40,91%	45,00%
	% row	60,00%	4,00%	18,00%	18,00%
	% whole sample	30,00%	2,00%	9,00%	9,00%
GG	Number	13	2	4	4
	% column	25,00%	33,33%	18,18%	20,00%
	% row	56,52%	8,70%	17,39%	17,39%
	% whole sample	13,00%	2,00%	4,00%	4,00%
AA	Number	9	2	9	7
	% column	17,31%	33,33%	40,91%	35,00%
	% row	33,33%	7,41%	33,33%	25,93%
	% whole sample	9,00%	2,00%	9,00%	7,00%
Total N		52	6	22	20
% whole		52,00%	6,00%	22,00%	20,00%
**DRD1 rs686**					
AA	Number	13	1	9	3
	% column	25,00%	16,67%	40,91%	15,00%
	% row	50,00%	3,85%	34,62%	11,54%
	% whole sample	13,00%	1,00%	9,00%	3,00%
AG	Number	29	4	10	13
	% column	55,77%	66,67%	45,45%	65,00%
	% row	51,79%	7,14%	17,86%	23,21%
	% whole sample	29,00%	4,00%	10,00%	13,00%
GG	Number	10	1	3	4
	% column	19,23%	16,67%	13,64%	20,00%
	% row	55,56%	5,56%	16,67%	22,22%
	% whole sample	10,00%	1,00%	3,00%	4,00%
Total N		52	6	22	20
% whole		52,00%	6,00%	22,00%	20,00%

Included patients were adults (age above 18 years); diagnosed with probable SB on the basis of clinical symptoms such as: the presence of masticatory muscle hypertrophy as well as indentations on the tongue or lip and/or a linea alba on the inner cheek, damage to the dental hard tissues (e.g., cracked teeth or tooth wear), repetitive failures of restorative work/prosthodontic constructions with or without positive self-reporting^4^.

Participants were excluded from the study if they presented secondary bruxism induced by systemic diseases, e.g., Parkinson’s disease; used medicines that can significantly affect the functioning of the nervous and muscular systems; presented severe mental disorders and severe systemic (including genetic) diseases; were unable to undergo polysomnography, due to severe mental retardation or Alzheimer’s disease; presented neurological disorders and/or neuropathic pain, respiratory insufficiency, or active inflammation; were treated with or addicted to analgesic drugs and/or drugs that affect muscle and breath function; and presented active malignancy.

All of the standardized overnight, single-night polysomnographic examinations made using Nox-A1 (Nox Medical, Iceland) took place in the Sleep Laboratory of the Department of Internal Medicine, Occupational Diseases, Hypertension and Clinical Oncology, Wroclaw Medical University, Poland.

Polysomnograms (PSGs) were assessed in 30-s epochs according to the American Academy of Sleep Medicine (AASM) 2013 standard criteria for sleep scoring. PSG outcome variables included sleep latency; total sleep time and sleep efficiency (%); the ratio of N1 (sleep stage 1), N2 (sleep stage 2), and N3 (sleep stage 3); and the stage of REM (rapid eye-movement sleep). Abnormal respiratory events were scored from the nasal pressure airflow signal evaluated according to the standard criteria of the American Academy of Sleep Medicine Task Force^58^. Apnea was defined as the absence of airflow for ≥10 s. Hypopnea was defined as a reduction in the amplitude of breathing by ≥30% for ≥10 s with ≥3% decline in blood oxygen saturation or an arousal.

Sleep bruxism was assessed by performing electromyography (EMG) of bilateral masseter muscles and by subsequent evaluation of video and audio recordings. Bruxism episodes were scored into three forms according to the AASM standards: phasic, tonic, and mixed. The AASM standards specify that for confirming the presence of SB, EMG activity has to be at least twice the amplitude of the background EMG, and EMG bursts should not have been separated by >3 s to be considered a part of the same episode. A constant burst episode sustained for over 2 seconds in masseter EMG recording was categorized as tonic, an episode including three or more bursts for over 2 seconds was categorized as phasic, and a combination of tonic and phasic episodes was categorized as mixed^59^.

The BEI measures the number of bruxism episodes per hour of sleep (<2: irrelevant SB; 2–4: mild/moderate SB; >4: severe SB)^60^.

The scoring of SB episodes and analysis of collected data were performed by a qualified physician (H.M.) from the Sleep Laboratory of the Department of Internal Medicine, Occupational Diseases, Hypertension and Clinical Oncology, Wroclaw Medical University, Poland.

In the studied group, DNA was extracted from peripheral blood samples drawn into ethylenediaminetetraacetic acid (EDTA)-containing tubes using the Qiagen DNA Isolation Kit (Qiagen GmbH, Hilden, Germany), following the recommendations of the manufacturer. The blood samples were collected again the next day after conducting the polysomnographic study. Then the samples were frozen at –20 °C until further use.

In the control group, DNA was extracted from peripheral blood samples collected into EDTA tubes using the Qiagen DNA Isolation Kit (Qiagen GmbH, Hilden, Germany), following the recommendations of the manufacturer. The samples were stored at –20 ^o^C until further use.

The study was approved by the Ethics Committee of the Wroclaw Medical University (no. KB-195/2017) and was conducted in accordance with the principles of Declaration of Helsinki. All patients gave written informed consent. The study was also registered in the international database for clinical studies (Trial registration: Clinical Trials NCT03083405, WMU1/2017, https://clinicaltrials.gov/ct2/show/NCT03083405).

### DNA isolation, SNP selection, and genotyping

The SNPs were selected on the basis of their functional roles. Both SNPs were located within the *HTR2A* gene (rs6313 and rs2770304) and were previously found to be associated with the risk for SB development in Japanese^13,33^ and Chilean^34^ patients, respectively. The *HTR2A* rs6313 is a synonymous T/C substitution in exon 1, while the rs2770304 SNP is located within intron 2. rs6316 and rs6311 polymorphisms in the promoter region are the most frequently studied SNPs within the *HTR2A* gene, and they are located 1538 bp apart on chromosome 13 and are in strong linkage disequilibrium^61^. Both these SNPs are associated with gene expression levels^29–31^. A previous post-mortem study of Turecki *et al*.^29^ has demonstrated that, compared with the *HTR2A* rs6313 *T* allele, the presence of *C* variant was related to reduced 5-HT binding to 5-HT 2A serotonin receptor in the superior frontal cortex. Another *in vitro* study reported that the *G* allele of the rs6311 SNP, which is in absolute linkage disequilibrium with the *C* allele of rs6313, has a potential to negatively modulate the *HTR2A* promoter activity^30^. More recently, another post-mortem brain study reported that the expression of 5-HT2A was higher in individuals carrying the *C* allele of rs6313 (or *G* allele of rs6311) than in individuals with the *TT* genotype^31^. The third of the studied SNPs is a non-synonymous G/A polymorphism within exon 4 of the *COMT* gene resulting in a Val158Met substitution. The fourth SNP, rs686, is located within the 3′UTR of the DRD1 encoding gene, and thus can be affected by microRNA molecules. This polymorphism modulates the regulation of the *DRD1* gene expression^57^, which is due to the fact that this SNP is located in the binding region of miR-504^56^.

All the four SNPs were studied in 100 study group patients and 125 controls except for *HTR2A* rs6313 SNP, which was genotyped in 99 patients.

The determination of the *HTR2A* (rs2770304 C > T and rs6313 C > T), *COMT* (rs4680 G > A), and *DRD1* (rs686 G > A) polymorphisms was carried out by the LightSNiP typing assay (TIB-MolBiol, Berlin, Germany), which included amplification by real-time polymerase chain reaction (PCR) and subsequent analysis of the melting curve. The reactions were performed in a LightCycler 480 II system (Roche Diagnostics, Rotkreuz, Switzerland) according to the manufacturer’s recommendations.

The LightSNiP assays incorporate SNP-specific probes and use real-time PCR amplifications followed by melting curve analysis, as previously described^62^. SNP variants are identified based on differences in DNA melting temperature. The reaction mix was composed of: 1.6 μL of MgCl2, 14.4 μL of H2O, 1 μL of the LightSNiP reagent, and 2 μL of FastStart DNA Master HybProbe (Roche Diagnostics), and the final volume was made up to 20 μL by adding 1 μL of DNA solution. The reaction program was as follows: initial denaturation at 95 °C for 10 min, followed by 45 cycles of denaturation at 95 °C for 10 s, annealing at 60 °C for 10 s, and extension at 72 °C for 15 s. The melting curve analysis was proceeded by a 30 s incubation in 95 °C, followed by 2 min in 40 °C. The actual melting analysis was performed with continuous acquisition of data in the range of 40–75 °C, with a ramp rate of 1.5 °C/s.

### Statistical analysis

Statistical analyses were conducted in two steps.

In the first step, we studied selected SNPs in the study group and in the control group. Chi-squared test was used to test for deviations from Hardy–Weinberg equilibrium. The Fisher’s exact test was employed to compare genotype and allele frequencies between patients and controls (calculated on the website http://vassarstats.net/tab2x2.html). P-values <0.05 were considered statistically significant^62^. In addition Odd’s Ratio (OR) values were calculated to assess the potential association of selected SNPs with susceptibility (OR > 0) or protection (OR < 0) for bruxism development.

In the second step, we investigated differences in the levels of clinical parameters between study group patients with various polymorphisms. Since the clinical data were non-normally distributed and did not satisfy the assumptions of parametric analyses, the nonparametric Kruskal–Wallis (K-W) tests were performed. One exception was BEI where log transformation reduced the skewness of the raw data and allowed to analyze the genotype differences by parametric one-way ANOVA. If the omnibus tests in K-W or ANOVA models were statistically significant, post-hoc multiple comparisons were conducted to further investigate differences between the analyzed genotypes (for K-W: Siegel and Castelan, 1988; for ANOVA: Tukey’s honest significant difference). The effect sizes for the statistically significant K-W models were calculated according to Cohen (2008). In this step of the analysis, we also studied the association between BEI and AHI scores in different groups of patients using Spearman’s rank correlation coefficients.

Clinical relevance of detected polymorphisms was assessed by sensitivity (i.e. the proportion of patients with the given condition having the given genotype) specificity (i.e. the proportion of patients without the given condition not having the given genotype), positive and negative predictive values (probability of having the given condition while having the given genotype and probability of being without the given condition while not having the given genotype respectively). All characteristics were calculated separately for OSA only, SB only and co-occurring SB and OSA patients diagnosed in the whole study group.

P-values <0.05 were considered to be statistically significant, and those between 0.05 and 0.10 were indicative of a trend (marginally significant). Analyses were carried out using STATISTICA software, version 12 (StatSoft, Inc., Tulsa, USA) and R (R Core Team, 2019)^63^.

## Data Availability

The datasets generated and/or analysed during the current study are available from the corresponding author on reasonable request.
